# Environmental factors influencing fine-scale distribution of Antarctica’s only endemic insect

**DOI:** 10.1007/s00442-020-04714-9

**Published:** 2020-07-29

**Authors:** Leslie J. Potts, J. D. Gantz, Yuta Kawarasaki, Benjamin N. Philip, David J. Gonthier, Audrey D. Law, Luke Moe, Jason M. Unrine, Rebecca L. McCulley, Richard E. Lee, David L. Denlinger, Nicholas M. Teets

**Affiliations:** 1 Department of Entomology, University of Kentucky, S-225 Agricultural Science Center North, Lexington, KY 40546 USA; 2 Department of Biology, Hendrix College, Conway, AR USA; 3 Department of Biology, Adolphus College Gustavus, Saint Peter, MN USA; 4 Department of Biology, Miami University, Oxford, OH USA; 5 Department of Plant and Soil Science, University of Kentucky, Lexington, KY USA; 6 Department of Entomology, Ohio State University, Columbus, OH USA

**Keywords:** Abiotic environment, Antarctic midge, Biotic influences, Spatial distribution

## Abstract

Species distributions are dependent on interactions with abiotic and biotic factors in the environment. Abiotic factors like temperature, moisture, and soil nutrients, along with biotic interactions within and between species, can all have strong influences on spatial distributions of plants and animals. Terrestrial Antarctic habitats are relatively simple and thus good systems to study ecological factors that drive species distributions and abundance. However, these environments are also sensitive to perturbation, and thus understanding the ecological drivers of species distribution is critical for predicting responses to environmental change. The Antarctic midge, *Belgica antarctica*, is the only endemic insect on the continent and has a patchy distribution along the Antarctic Peninsula. While its life history and physiology are well studied, factors that underlie variation in population density within its range are unknown. Previous work on Antarctic microfauna indicates that distribution over broad scales is primarily regulated by soil moisture, nitrogen content, and the presence of suitable plant life, but whether these patterns are true over smaller spatial scales has not been investigated. Here we sampled midges across five islands on the Antarctic Peninsula and tested a series of hypotheses to determine the relative influences of abiotic and biotic factors on midge abundance. While historical literature suggests that Antarctic organisms are limited by the abiotic environment, our best-supported hypothesis indicated that abundance is predicted by a combination of abiotic and biotic conditions. Our results are consistent with a growing body of literature that biotic interactions are more important in Antarctic ecosystems than historically appreciated.

**Electronic supplementary material:**

The online version of this article (10.1007/s00442-020-04714-9) contains supplementary material, which is available to authorized users.

## Introduction

Species distributions and population sizes are dependent on interactions among physical, chemical, and biological factors (Hughes et al. 1997; Westgate et al. 2014). Abiotic factors that regulate species distribution include climatic features such as temperature, moisture and availability of macro- and micronutrients (Guisan and Thuiller 2005), whereas biotic influences include intra- and interspecific interactions, life history traits, and demography (Chong et al. 2015). Identifying the important ecological factors that drive community structure is fundamental for understanding the effects of biodiversity on ecosystem functioning (Caruso et al. 2019; Lee et al. 2019). Further, with biodiversity being threatened by climate change and other human impacts, understanding the influence of abiotic and biotic factors on species distribution is critical for predicting responses to environmental change (Bokhorst et al. 2019).

The habitats of terrestrial maritime Antarctica are among the most extreme yet simplified ecosystems on our planet. The simplicity of these ecosystems allows for thorough cataloging of the abiotic and biotic components of an ecosystem, which is not often possible in other locations. Antarctic habitats are characterized by intense abiotic stressors, including low temperature, limited water availability and variable nutrient levels (Convey 1997), and these ecosystems have significantly lower biodiversity than the rest of the planet (Convey and Stevens 2007). For example, while more than one million species of insects have been described worldwide, only three are found on the continent of Antarctica. While several studies have characterized terrestrial species assemblages in Antarctic ecosystems (Convey et al. 2008; Schlensog et al. 2013), few have identified factors that underlie variation in community composition and abundance of particular species (Chown and Convey 2016). In contrast to temperate and tropical environments, biotic interactions such as competition, parasitism, and predation have historically been predicted to be less important for species distribution and population size in Antarctica. Instead, abiotic factors such as temperature and water availability have been considered the primary drivers of terrestrial communities (Kennedy 1993; Convey 1996; Hogg et al. 2006; Sinclair et al. 2006; Bissett et al. 2014; Chown and Convey 2016). However, recent evidence challenges these long-held hypotheses and indicates an important role for biotic interactions in shaping Antarctic communities.

Recent work in Antarctica indicates a trophic cascade in which nitrogen deposition by penguin and seal colonies supports cryptogam abundance and quality, which in turn supports microarthropod abundance (Bokhorst et al. 2019). Plant life in these regions tends to be nitrogen limited (Wasley et al. 2006), so this fertilization of ice-free areas by marine vertebrates is critical for terrestrial plants and arthropods. Bokhorst et al. (2019) also showed that water availability and temperature, which were historically considered the limiting variables for Antarctic terrestrial organisms, are not strong predictors for arthropods in these study areas. Even in the McMurdo Dry Valleys, which contain the driest and coldest soils on Earth, community assemblages of microbes and nematodes are influenced by both abiotic factors and biotic interactions (Caruso et al. 2019; Lee et al. 2019). Thus, despite having reduced biodiversity, direct and indirect interspecific interactions appear to be important drivers of terrestrial Antarctic communities.

The terrestrial arthropod communities of Antarctica are relatively depauperate, limited to three true insects, approximately 15 species of Collembola, and 50 species of free-living mites (Convey 2011). Of these, the midge *Belgica antarctica* is Antarctica’s only endemic insect and is found exclusively on offshore islands along the west coast of the Antarctic Peninsula. First described in 1900 (Jacobs 1900), the basic life history and physiology of this midge are well studied (Lee and Denlinger 2014). Adult midges are brachypterous and emerge for a brief period during the austral summer to mate and lay eggs, and larvae require 2 years to complete their life cycle. Larvae are typically associated with algae, moss and grass, and are often found in nutrient-enriched soils near nesting birds and seal wallows (Peckham 1971). There is considerable variation in midge distribution and abundance across islands. Rico and Quesada (2013) surveyed the abundance of *B. antarctica* on Byers Peninsula in maritime Antarctica and found an average of 193 larvae/m^2^ in dry littoral areas, while density reached 42,000 larvae/m^2^ in moist, moss-heavy sites. However, the precise factors that regulate this considerable fine-scale variation in midge abundance have not been quantified. From a previous study of arthropods (Collembola and mites) in maritime Antarctica, variables that influence abundance were dependent on the spatial scale investigated, even at relatively small spatial scales overall (Usher and Booth 1986). At spatial scales between 45 and 70 cm, abiotic environmental variables including moisture content were correlated to arthropod abundance, while at smaller scales (5–10 cm), there were strong correlations between the abundances of different species, suggesting that distinct taxa share similar habitat requirements (Usher and Booth 1986). Recent work on nitrogen deposition in terrestrial Antarctica indicates that the influence of marine vertebrates can span several kilometers (Bokhorst and Convey 2016), but whether nitrogen limitation regulates fine-scale distribution has not been investigated. Thus, studies operating at distinct spatial scales are needed to advance understanding of the ecological drivers of Antarctic arthropod distribution.

The habitats of *B. antarctica* have relatively simple community composition. Midges are often found in close proximity to other terrestrial arthropods, specifically Collembola and mites. Interspecific competition or mutualism between these species has not been directly observed, and *B. antarctica* also does not have any known predators, so interactions with terrestrial animals appear to be limited (Chown and Convey 2016). Primary producers including cryptogams (moss and the algae *Prasiola crispa*) and a single grass species, *Deschampsia antarctica* Desv. (Poaceae) are thought to be important regulators of midge abundance, as larvae of *B. antarctica* are non-selective feeders that consume terrestrial algae and decaying plants (Strong 1967; Peckham 1971; Baust and Edwards 1979). The primary abiotic factors that regulate distribution and abundance of *B. antarctica* and other arthropods are likely temperature and moisture content (Sinclair and Sjursen 2001; Sinclair et al. 2006), as ecologically relevant temperature and water stress can cause mortality and sublethal injury (Teets and Denlinger 2014). Nutrient availability is another factor that likely influences midge abundance, as terrestrial Antarctic habitats are often nitrogen limited (Bokhorst and Convey 2016; Bokhorst et al. 2019). Beyond our interests in this single extreme-adapted species, the simplicity of Antarctic ecosystems, coupled with their considerable habitat heterogeneity, makes these areas excellent systems to identify environmental factors that regulate fine-scale species distribution.

Here, we quantify the relative contribution of abiotic and biotic factors on the local distribution of *B. antarctica*. We sample midge larvae from five islands around Palmer Station, which were chosen based on their diversity of habitat types and presence of key ecological features, described below. We collect data on both abiotic and biotic factors at all sites and test five hypotheses to identify the important factors regulating midge abundance:

### Hypothesis 1: bulk composition of the substrate regulates midge abundance

Water content of the substrate has been historically considered the most important factor determining arthropod abundances in Antarctic ecosystems (Kennedy 1993; Sinclair and Sjursen 2001), and macronutrient composition is also important on broad spatial scales (Bokhorst et al. 2019). Our first hypothesis tests for the effect of these bulk components of the substrate by testing the effect of substrate carbon, nitrogen, and water content.

### Hypothesis 2: substrate micronutrients regulate midge abundance

While macronutrients and water content appear to play an important role in regulating arthropod abundance, the contribution of micronutrients and other elemental substances to Antarctic arthropod distribution has not been addressed. Aluminum and sodium are positively associated with moss abundance (Ino and Nakatsubo 1986; Fabiszewski and Wojtun 2000), indicating minor elements of the soil may play an important role in these ecosystems. Our second hypothesis tests for the effect of low abundance components of the substrate, including aluminum, calcium, iron, magnesium, phosphorous, potassium, sodium, sulfur, and pH (which can be affected by the abundance of some of these micronutrients).

### Hypothesis 3: biotic interactions are important regulators of midge abundance

There is growing literature that biotic interactions are more important in Antarctica than previously appreciated, and our third model includes plant and arthropod abundance to test whether biotic interactions influence fine-scale midge distribution. Larvae of *B. antarctica* consume the algae *Prasiola crispa* and decaying plants, so we predict that plant abundance will be positively associated with midge abundance. Previous work indicates that *B. antarctica* and other terrestrial arthropods have similar habitat requirements (Chown and Convey 2016), so we predict a positive relationship between midges and other terrestrial arthropods.

### Hypothesis 4: the combined effects of nitrogen and biotic interactions predict midge abundance

Recent studies on arthropod abundance in Antarctic ecosystems indicate a bottom-up trophic cascade regulated by nitrogen deposition from marine vertebrates (Bokhorst et al. 2019). While we did not directly test for a trophic cascade, our fourth hypothesis includes both nitrogen and biotic factors to test whether the inclusion of both improves our predictions of midge abundance.

### Hypothesis 5: the combined effects of abiotic and biotic interactions predict midge abundance

While Antarctic habitats have been historically considered to be abiotically regulated, recent evidence indicates that Antarctic community structure is shaped by both abiotic factors and biotic interactions (Lee et al. 2019). Previous work in *B. antarctica* has not addressed both components simultaneously, but for other soil invertebrates, bacteria, and fungi, the inclusion of both abiotic and biotic variables improves statistical models of soil communities (Lee et al. 2019). In this hypothesis, we include all variables to test for the combined influences of abiotic and biotic effects on midge abundance.

## Methods

This study was conducted on offshore islands in the vicinity of Palmer Station, which is located on Anvers Island on the Antarctic Peninsula (64°46′S, 64°04′W). All samples were collected in January 2018 and either processed immediately or shipped frozen to the University of Kentucky for analysis.

### Transects

**Fig.1:**
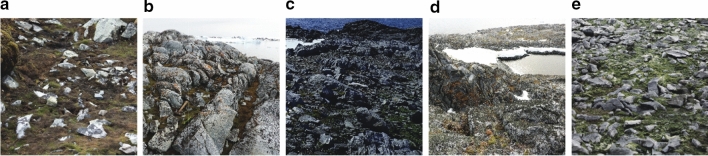
Photographs of the five collecting sites where we established transects. Amsler Island (**a**), Christine Island (**b**), Cormorant Island (**c**), Joubins Island 4 (**d**) and Torgersen Island (**e**)

Samples were collected from Amsler Island (64°45′S, 64°05′W), Cormorant Island (64°47′S, 63°57′W), Torgersen Island (64°46′S, 64°05′W) Christine Island (64°43′S, 64°13′W), and Joubins Island 4 (64°47′S, 63°57′W). These islands are established collecting sites for midges, and they show considerable variation in vegetation and geology that reflects the range of habitats typically occupied by midges in maritime Antarctica. Because our goal was to assess quantitative variation in midge abundance, we began transects at midge collecting sites known from previous collecting trips (Fig. 1). A brief description of the island transects is as follows: (1) Amsler Island: a deep moss bed on a ridge, approximately 5 m above sea level; (2) Cormorant Island: a gradual rocky slope dominated by moss and algae, and the transect was approximately 15 m from a small Adelie penguin colony and ended approximately 10 m from shore; (3) Torgersen Island: a flat algae-covered rock bed, within 15 m of a large Adelie penguin colony; (4) Christine Island: an abandoned seal rookery approximately 20 m from shore; (5) Joubins Island 4: a steep elevational gradient, starting about 20 m from the shore in an area with little vertebrate animal activity, with the exception of scattered skua nests. At each island, our transect began at a known collecting site with high midge density, and from there a 20 m line was followed that avoided large rocks and bare patches with no vegetation. By sampling outward from a known area of high midge density, we increased our chances of including plots that spanned the range of densities found within a typical habitat. Samples were collected at 1 m intervals, and at each plot, we placed a 900 cm^2^ square that was divided into nine 100 cm^2^ areas, and the following abiotic and biotic variables were measured.

### Abiotic variables

Moisture content of the substrate was calculated by taking a 1–2 g piece of substrate from the sampled area and drying in an oven for 12 h at 60 ℃. Samples were weighed before and after drying to obtain percent moisture content.

Carbon and nitrogen contents were analyzed from substrate samples with arthropods removed. At each site, one 50 ml tube of substrate was reserved, stored on station at − 80 °C, and shipped to the University of Kentucky on dry ice. At the University of Kentucky, samples were analyzed for total carbon and nitrogen on a FlashEA 1112 elemental analyzer (ThermoFisher Scienitifc Inc., Waltham, MA). Elemental composition (aluminum, calcium, iron, magnesium, phosphorous, potassium, sodium, and sulfur) was also measured on the same substrate samples. For elemental composition, samples (up to 250 mg dry powder) were microwave digested (CEM MARSXpress, Matthews, NC, USA) in 10 mL trace-metal grade nitric acid in sealed Teflon bombs at 180 ℃ for 10 min following EPA method 3052. Elemental content was determined using inductively coupled plasma optical emission spectrometry (ICP-OES; Agilent 5110 SVDV, Santa Clara, CA) following EPA method 6010d. To assess quality control, procedural blanks, cross calibration verification, duplication, and spike recovery were performed as outlined in EPA method 6010d. We also assessed recovery of acid extractable elements from a standard reference material (National Institute of Standards and Technology SRM 2709, San Joaquin Soil, Gaithersburg, MD, USA). Substrate pH was measured by combining 1 g substrate with 1 ml water, and the pH of the resulting slurry was measured with an electronic pH reader (Orion 3-Star pH Benchtop).

### Biotic variables

At each plot, we counted the number of midges (*B. antarctica*), Collembola (*Cryptopygus antarcticus*), and mites (*Alaskozetes antarcticus*) and expressed these counts as density per m^2^. In a preliminary assessment, we determined that most arthropods were found within 1.5 cm of the surface at these sites. To ensure samples could be processed during our limited time on station, from each 900 cm^2^ plot, we collected arthropod samples from two adjacent squares in our 3 × 3 sampling grid (a total area of 200 cm^2^), rather than sampling the entire 900 cm^2^ plot. We collected the top 1.5 cm of substrate (moss, algae, grass, and rocks) and placed it in a plastic bag. Substrate was returned to Palmer Station, and arthropods were extracted by spreading substrate on a mesh screen and using a halogen lamp to coerce arthropods into a collecting pan of ice water below. These samples were then hand counted and sorted by taxa, and the counts were converted to densities per square meter. Previous collecting experience indicated some degree of spatial separation between midges and other arthropods, likely owing to the limited dispersal ability of these species. Our samples are biased towards midge habitats, so Collembola and mite densities were likely not a true representation of their potential values. We still included them as a biotic variable in our model to test for any associations with midges. Microhabitat composition of each plot was assessed by calculating percent cover of moss, algae, and grass from the nine squares.

### Statistical models

Of the 100 plots sampled (5 islands, 20 plots per island), 11 were excluded because of incomplete data fields, and we wanted to have a complete dataset for the modeling. Final sample sizes for the five islands were: Amsler (*n* = 13), Christine (*n* = 19), Joubins (*n* = 20), Torgersen (*n* = 18) and Cormorant (*n* = 19).

To test our five hypotheses (see Introduction), we fit generalized linear mixed models using ‘PROC GLIMMIX’ in SAS v.9.4. In total, we fit the following models: Model 1: midge density as a function of percent nitrogen, carbon and moisture of the substrate; Model 2: midge density as a function of total amount of aluminum, calcium, iron, magnesium, phosphorous, potassium, sodium, sulfur, and pH of the substrate; Model 3: midge density as a function of Collembola and mite density, and percent cover of moss, algae, and grass; Model 4: midge density as a function of nitrogen content, Collembola and mite density, and percent cover of moss, algae, and grass (Table 1). Model 5: A full model containing all abiotic and biotic variables. In addition, we also fit a null model for comparison. For each model, we calculated the variance inflation factor (VIF) for each variable using function PROC REG in SAS. Variables were considered for collinearity by excluding variables with VIF > 15, then VIF > 10, then VIF > 5. Thus, the final variables left in the models all had VIF < 5. Based on VIF results, the following variables were excluded from each model: Model 1: none, all variables were included; Model 2: calcium, iron, phosphorous, potassium; Model 3: none, all variables were included; Model 4: none, all variables were included. Model 5: carbon, calcium, iron, magnesium, phosphorous, potassium, and sodium. For all models, midge densities were square root transformed to ensure normality of the model residuals. Due to the influence of extreme values and an abundance of zero values in some independent variables, we elected to discretize the independent variables into “high” (> 50th percentile) and “low” (< 50th percentile) categories. Island was treated as a random effect in the models. Spatial correlation structure among the residuals was accounted for by modeling island-specific spatial power covariance functions using the distance between transects on each island as the distance function, using the proc GLIMMIX function where *x* is the distance from the first plot in a transect on a particular island (Stroup 2012). To determine the best-supported hypotheses, we compared models using AIC values.

**Table 1:** Summary of modeling results to test hypotheses on the contribution of abiotic and biotic environmental variables on midge abundance

	AIC		Estimate	*P* value
Null model	930			
Model 1: Bulk composition	909	Nitrogen	4.09	0.684
		Carbon	− 16.96	0.110
		Moisture	− 14.97	0.089
Model 2: Elemental substances	893	pH	− 3.17	0.775
		Na	− 4.72	0.639
		Mg	− 7.03	0.448
		Al	13.35	0.101
		S	− 19.15	0.085
Model 3: Plants and arthropods	892	Moss	2.89	0.820
		Grass	− 4.95	0.746
		Mite	− 7.93	0.625
		Collembola	8.80	0.493
		Algae	32.33	0.048
Model 4: Nitrogen influences midge and plants	885	Moss	5.49	0.676
		Mite	− 6.51	0.667
		Grass	− 7.25	0.647
		Collembola	8.38	0.511
		Nitrogen	8.33	0.434
		Algae	30.79	0.064
Model 5: Combined abiotic and biotic features	848	Nitrogen	− 4.83	0.667
		Moisture	− 20.33	0.028
		pH	− 2.17	0.838
		Al	14.75	0.086
		S	− 27.89	0.005
		Collembola	2.71	0.828
		Mite	− 13.59	0.383
		Moss	14.86	0.279
		Grass	− 11.32	0.480
		Algae	24.77	0.143

Spatially autocorrelated linear models were fit for each hypothesis, with island as a random effect. AIC values were used to compare our different hypotheses-driven models from a null model. The variables within each model are shown after removal of variables with high multicollinearity (VIF > 5). Model coefficients and *P* values are shown for each variable

Additionally, we used a principal components analysis (PCA) and pairwise correlations to further assess the structure of our dataset. The PCA was used to cluster plots by similarity and determine which variables were most responsible for separation among plots. This analysis was completed in R using the ‘prcomp’ function with scaling and graphed using the ‘ggbiplot’ package. A correlation matrix was calculated in R using the package ‘corrplot’ and graphed using ‘ggcorrplot’. For PCA and correlation analysis, we used square-root-transformed values of midge, Collembola and mite densities, and spatial correlation among plots was not included in these exploratory analyses.

## Results

### Midge population density

**Fig. 2:**
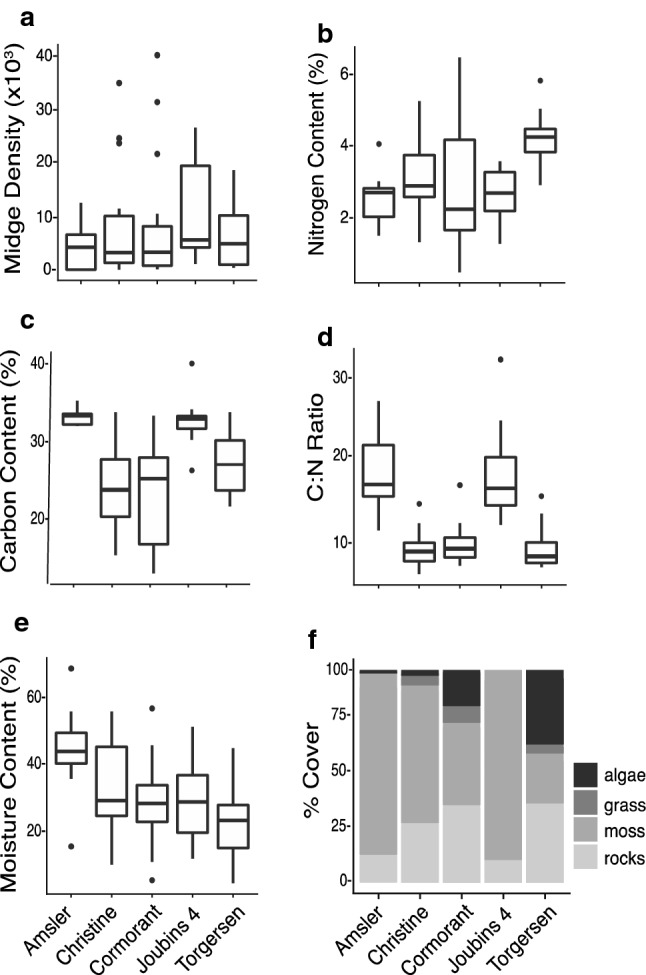
Box and whisker plots showing variation in midge abundance and habitat characteristics on each island. **a** Midge abundance, **b** Percent nitrogen of substrate, **c** Percent carbon of substrate (**c**), **d** C:N Ratio of substrate, **e** Percent moisture content of substrate, and **f** Distribution of habitat substrate types. In (**f**), the stacked bar chart shows the average proportion of moss, algae, grass, and rock cover for each island

Across the five islands, there was variation in midge density, although within-island variation was considerably larger than between-island variation (Fig. 2a). Joubins Island had the highest median midge density of 5400/m^2^ (range = 1050–25,700/m^2^), while Amsler Island had the lowest, with 4050/m^2^ (range = 0–12,100/m^2^). The maximum density of midges collected at one plot was 38,850/m^2^, collected from Cormorant Island.

### Abiotic variables

Percent nitrogen and carbon was analyzed, and the C:N ratio was calculated. Nitrogen content differed across islands (Fig. 2b), with substrate from Torgersen Island having the highest median nitrogen content of 4.23% (range = 2.92–5.77%), and substrate from Cormorant Island having the lowest median nitrogen content of 2.26% (range = 0.54–6.40%). Carbon content also differed across islands (Fig. 2c), with substrate from Amsler Island having the highest median content of 44.63% (range = 41.94–48.53%), and substrate from Christine Island having the lowest median content of 25.52% (range = 8.69–45.49%). The C:N ratio differed across islands (Fig. 2d), with Amsler Island having the highest median ratio of 16.51 (range = 10.42–27.60), and Torgerson Island having the lowest median ratio of 6.98 (range = 5.55–14.97). Amsler, and Joubins Islands, which had the highest percentage of moss, tended to have the highest carbon and lowest nitrogen levels. Islands with high levels of vertebrate activity, i.e. penguin rookeries and elephant seal wallows, like Torgersen and Cormorant, had higher nitrogen and lower carbon.

Moisture content varied across islands (Fig. 2e), with substrate from Amsler Island having the highest median moisture content of 44% (range = 13–71%) and substrate from Torgerson Island having the lowest median moisture content of 22% (range = 1–45%). The pH of the substrate was highest on Christine Island with median of 5.94 (range = 3.92–7.33) and lowest on Amsler Island with median of 4.34 (range = 3.84–5.52). The micronutrient content of the substrate also differed across islands, and values are provided in Table S1.

### Biotic variables

In addition to midges, we also measured density of Collembola (*Cryptopygus antarcticus*) and mites (*Alaskozetes antarcticus*). Collembola density varied across islands, with Christine Island having the highest median density (2250/m^2^; range = 0–294,300/m^2^). Mite density also varied across islands, with Christine Island once again having the highest density of mites, with a median mite density of 2,400/m^2^ (range = 0–69,950/m^2^).

To compare microhabitats across sites, we calculated the percent cover at each plot for moss, algae, and grass (Fig. 2f). Moss cover was highest on Joubins Island with median coverage of 97% (range = 56–100%) and lowest on Torgerson Island with median coverage of 0% (range = 0–78%). Algae cover was highest on Torgerson Island with median coverage of 36% (range = 0–78%) and lowest on Joubins Island with no recorded algae cover. Grass was relatively rare in our plots, but grass was most abundant on Cormorant Island with a range of 0–44% (the median was 0%) and lowest on Amsler and Joubins Islands with no recorded grass cover.

### Ecological drivers of midge abundance

From the literature, we developed five hypotheses to explain variation in midge density across our plots. Using a generalized linear mixed model that accounts for spatial autocorrelation, we compare the AIC of our models to a null model for midge density (Table 1). Each of our models had considerably lower AIC values than the null (DAIC ≥ 21), indicating that all models were an improvement over the null model. Model 1, testing for the bulk abiotic composition of substrate had the highest AIC value of 909. Models 2 (substrate micronutrients) and 3 (biotic factors) had similar AIC value (893 and 892), Model 4, which combined nitrogen and the biotic factors, had an AIC of 885, and Model 5, which combined all abiotic and biotic variables had the lowest AIC of 848 despite including the most predictors. Across the five models, few terms were strong predictors of midge abundance. In Model 5, sulfur content was the strongest predictor of midge density (*T*_4,74_ = − 2.88, *P* = 0.0052) followed by substrate moisture (*T*_4,74_ = − 2.24, *P* = 0.0283). Both sulfur and moisture content were negative predictors of midge density. In all models with algae (models 3–5), the effect of algae on midge density was positive, indicating the higher algae cover resulted in higher midge densities. In Model 3, algae cover had a significant effect on midge abundance (*T*_4,79_ = 2.01, *P* = 0.0479), while in Model 4, algae was marginally significant. (*T*_4,78_ = 1.88, *P* = 0.0639). Although the effect was not significant in the Model 5, sites with high algae had an increase in midge density of approximately 600–1000/m^2^ relative to low density sites across Models 3–5.

### Multivariate analyses

**Fig. 3:**
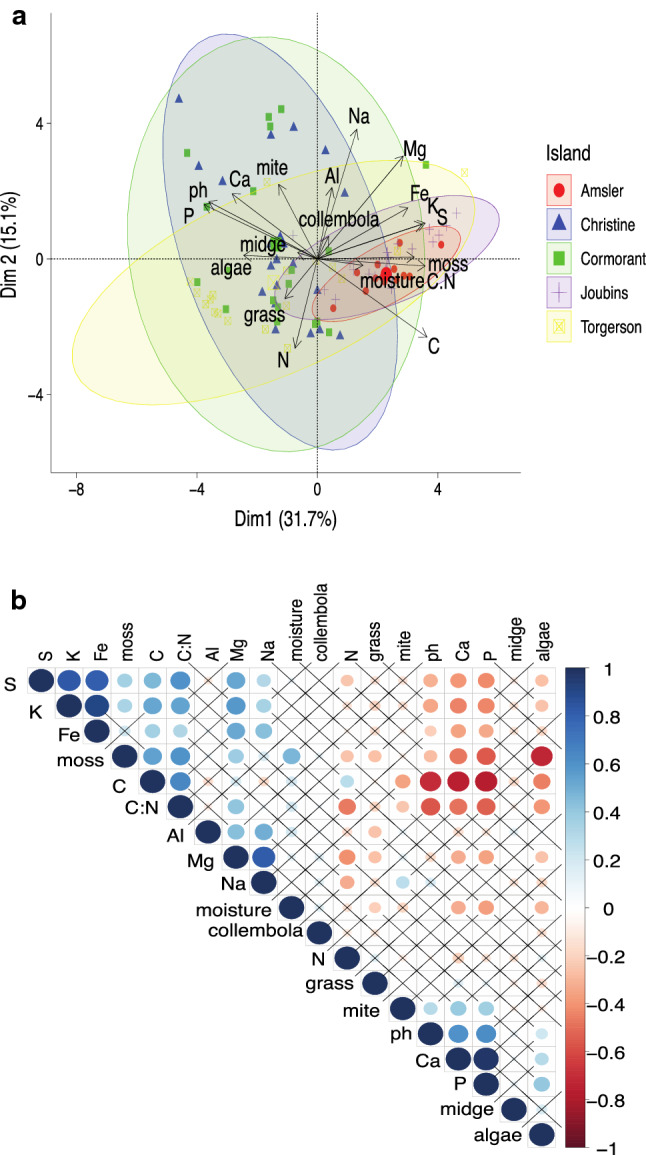
Principal component analysis and correlation matrix of all abiotic and biotic variables. **a** Principal components analysis, grouped by island, in which Dimension 1 explains 31.7% of the variation in our data set and Dimension 2 explains 15.1% of the variation. Shaded ellipses around plots are colored by island. **b** Correlation matrix of all variables. Blue indicates significant positive correlation, while red indicates significant negative correlation. Boxes that contain “X” are not statistically significant at *α* = 0.05

From our PCA, we determined which variables contributed most to the variance structure of our dataset (Fig. 3a). While samples from different islands showed significant overlap, samples from Amsler and Joubins Islands tended to cluster together and separate from the rest of the islands along PC1, which explained 31.7% of the variance in the dataset, and this separation was primarily driven by difference in phosphorous, calcium and carbon. PC2, which explained 15.1% of the variance in the dataset was primarily driven by sodium, magnesium, and nitrogen. Algae were aligned positively with midge density, consistent with it being the only significant positive predictor of midge density in our models.

To further visualize correlations in our dataset, we constructed a correlation matrix, which illustrates that many of the micronutrients are correlated with one another (Fig. 3b). This result is consistent with our VIF analysis in Models 2 and 5, where a majority of the micronutrients had a VIF > 5. Midge density had no significant pairwise correlations. Midge density had a marginally significant correlation with algae (*R* = 0.176, *P* = 0.098) and carbon was the highest negative correlation relationship (*R* = − 0.165, *P* = 0.122).

## Discussion

Species distribution and abundance depend on a variety of factors, including chemical, physical, and biological variables (Hughes et al. 1997; Westgate et al. 2014). We investigated the environmental factors that regulate insect abundance in a unique Antarctic ecosystem characterized by significant abiotic and biotic heterogeneity. Temperature and water have been historically considered the limiting variables in terrestrial Antarctica (Kennedy 1993; Convey 1996; Hogg et al. 2006; Sinclair et al. 2006; Bissett et al. 2014; Chown and Convey 2016), while more recent work indicates an important role for biotic interactions (Bokhorst et al. 2019; Caruso et al. 2019; Lee et al. 2019). Our focal species, the Antarctic midge, *Belgica antarctica,* has significant fine-scale variation in population density, but the factors that drive this patchy distribution have not been described. Here, we quantified the relative influence of abiotic and biotic factors on midge abundance across five islands on the Antarctic Peninsula. These islands vary considerably in vegetation and substrate characteristics, yet all had similar distributions of midge abundance characterized by dramatic fine-scale variation (Figs. 1 and 2). We generated five hypotheses to explain this fine-scale variation in midge abundance, and while all were improvements over a null model, the best-supported model included the full set of abiotic and biotic factors as predictors. These results are important for understanding the ecological drivers of fine-scale variation in population density and predicting responses of these communities to environmental changes.

### A combination of abiotic and biotic factors best predicts midge abundance

Our first three models separately examined the effects of abiotic and biotic factors on midge abundance. Models 1 and 2 tested for major and minor components of the abiotic environment, while Model 3 included biotic variables. Models 1–3 were more supported than the null, but based on AIC, Model 4 and particularly Model 5, which included the combined effects of abiotic and biotic variables, had the best support. Thus, fine-scale variation in midge abundance appears to be regulated by a complex interaction between abiotic and biotic environmental features. Model 4, informed by recent work by Bokhorst and Convey (2016); Bokhorst et al. (2019) included nitrogen and biotic factors, while Model 5 included all of the abiotic factors (with the exception of those removed after VIF analysis) and biotic components. Our most complex model, Model 5, was clearly the best supported based on AIC, and the remainder of the Discussion will primarily focus on this model.

The strong fit of Model 5 is consistent with recent research in Antarctica that indicates important roles for both biotic and abiotic conditions in shaping community composition. For example, in the Dry Valleys of Antarctica, which are severely water limited, it was originally presumed that soil communities were predominantly dependent on abiotic characteristics of the soil (Hogg et al. 2006). However, recent analyses indicate that the most abundant nematode species can negatively influence the abundance of less common species, suggesting that biotic interactions are important even in these relatively simple ecosystems (Caruso et al. 2019). Similarly, a recent study of the Dry Valleys examining a wide range of taxa, including bacteria, fungi, and multicellular invertebrates, again showed the importance of including both abiotic and biotic factors to explain species distributions (Lee et al. 2019). In this case, structural equation models that incorporated both abiotic conditions and linkages across functional groups were the best at explaining community structure (Lee et al. 2019). While we were unable to directly test directional linkages between nutrients, plants, and arthropods, our results are consistent with the growing body of evidence that including abiotic and biotic conditions are necessary for optimizing predictive models of soil community structure in Antarctica.

Based on recent work in Antarctica, we predicted that nitrogen would be an important predictor of fine-scale variation in midge abundance. In areas with adequate moisture, nitrogen fertilization by marine vertebrates promotes plant growth, and arthropods that depend on these plants show a similar positive association with nitrogen fertilization (Ryan and Watkins 1989; Bokhorst et al. 2019). However, in all of our models that included nitrogen as a predictor (Models 1, 4 and 5), nitrogen content was not a significant predictor of midge abundance (Table 1), despite a nearly 12-fold variation in nitrogen across out plots. Interestingly, the range of nitrogen values in our dataset was in line with those reported by Bokhorst et al. (2019) over a much larger area, who observed a strong effect of nitrogen on arthropod communities. This discrepancy suggests that that the specific chemical composition of the nitrogen, in addition to its abundance, may be important for arthropod abundance, or that there are species specific requirements for nitrogen (Bokhorst et al. 2019 did not assess midges). Also, this result highlights that predictors of species distribution may depend on the scale examined; while nitrogen availability is an important regulator of arthropod abundance over broad scales, we did not observe the same pattern for fine-scale variation in midge abundance.

In our best-supported model, substrate moisture content had a negative effect on midge abundance, with the wettest sites predicted to have approximately 400 fewer larvae per m^2^ (Table 1; *P* = 0.0283). These results were contrary to our expectations, as water is often considered the primary limiting factor for terrestrial life in Antarctica (Kennedy 1993; Convey, 1996; Sinclair and Sjursen, 2001; Hogg et al. 2006; Sinclair et al. 2006; Chown and Convey 2016).

Further, in previous quantitative sampling efforts for *B. antarctica,* the highest densities were found in areas with more moisture (Rico and Quesada 2013). This discrepancy can likely be explained by our sampling design. We established transects in areas of known midge abundance, so within these small areas, it is likely that water is not limiting. Instead, areas with high water content may have been waterlogged or flooded, as some of our plots were downslope from rapidly melting snow. Thus, in these water-saturated areas, it may be difficult for midges to establish, as overhydration caused by immersion in water can cause cellular stress (Lopez-Martinez et al. 2009). While water is a limiting factor for midges over broad scales, within these small transects, we observed the opposite pattern, suggesting that too much water can be detrimental within otherwise suitable habitats.

The other significant negative predictor of midge abundance was sulfur, as areas with high sulfur were predicted to have nearly 800 fewer larvae per square meter than low sulfur plots (*P* = 0.0052; Table 1). Sulfur is an essential nutrient for living organisms, primarily as a component of the amino acids methionine and cysteine, and it is mobilized by mineral weathering, where it accumulates in the soil and is taken up by plants and soil microorganisms (Barrett et al. 2007). In Antarctica, with limited plant and microbial activity, the abundance of sulfur in the environment is influenced by soil microorganisms and, where present, plants and or seabirds (Prietzel et al. 2019). In the Antarctic Peninsula, areas with higher moss colonization also have higher sulfur content, presumably due to sequestration by moss tissue (Prietzel et al. 2019). Additionally, sulfur is often positively associated with soil salinity, and in East Antarctica, most invertebrates are found in lower abundance in high sulfur, high salinity areas (Czechowski et al. 2016). Indeed, in our samples, sulfur and sodium were positively correlated (*R* = 0.302, *P* = 0.0039, Fig. 3b), indicating that the relationship between midges and sulfur may be similar to that observed for invertebrates in East Antarctica.

While sulfur is an essential mineral for life, the negative relationship between sulfur and midge abundance may be driven in part by differences in sulfur composition of various plants. Sulfur was negatively correlated with algae coverage (*R* = − 0.27, *P* = 0.012, Fig. 3b) and across our models, algae was a positive predictor of midge abundance. This result is consistent with previous records of algae (specifically *Prasiola crispa*) being a preferred food source for *B. antarctica* (Convey and Block 1996; Chown and Convey 2016; Peckham 1971). In contrast, sulfur was positively correlated with moss coverage (*R* = 0.35, *P* = 0.0007; Fig. 3b) which is likely explained by sulfur accumulation in moss, discussed above (Prietzel et al. 2019). While moss cover was not strongly associated with midge abundance in our plots, our analyses did not account for the growth stage of the moss. Anecdotally, we typically find midges in decaying moss rather than dense new growth, which is consistent with earlier claims that midges are incapable of feeding on live moss tissue (Convey and Block 1996). Thus, actively growing moss, which is also actively accumulating sulfur, may be incompatible with midges and partly explain the observed negative relationship between midge abundance and sulfur content.

### Extreme fine-scale variation in midge abundance

A final noteworthy result from our study is the extent of variation in midge density across our sites. Across our 100 plots, midge density ranged from 0 to 38,850 larvae/m^2^, with high variation among plots. For instance, on Cormorant Island, density in the 20 plots (each separated by 1 m) ranged from 50 to 38,850 larvae/m^2^. In some cases, even adjacent plots had considerable variation in density; for example, the highest density plot on Cormorant (38,850 larvae/m^2^) was adjacent to a plot with 3150 larvae/m^2^. However, the environmental conditions of these plots were similar, at least relative to the distribution of conditions across all plots; for example, both plots had relatively high algae cover (56% and 78%), and substrate moisture levels were intermediate for that island (28% and 19%). Thus, while our modeling efforts indicate that a combination of abiotic and biotic factors best describes variation in midge abundance, we were unable to completely capture the complexity of these systems. We speculate that temporal variation in conditions may play an important role in these environments, and our sampling was a single snapshot in time. Also, we suspect that selection of oviposition sites by females may be an important determinant of midge distribution. Females lay egg masses containing 30–65 eggs (Lee and Denlinger 2014), while larvae have a limited (or no) ability to disperse. However, the factors that determine selection of oviposition sites are unknown. Thus, despite our efforts to comprehensively quantify the abiotic and biotic environment, additional work is needed to completely characterize the factors that result in the extreme patchy distribution of *B. antarctica.*

## Conclusions

Relative to other ecosystems, the simplicity of Antarctic ecosystems facilitates in-depth studies of community composition, and understanding these ecological relationships is critical for predicting how these sensitive habitats will respond to environmental change. This study includes the most comprehensive assessment of microhabitat conditions that influence local variation in population sizes for an Antarctic terrestrial arthropod. Previous studies suggest that moist, nutrient-enriched soils that promote plant cover are required for Antarctic arthropods (Convey and Block 1996; Rico and Quesada 2013; Chown and Convey 2016), and more recently, it was demonstrated that nitrogen input from large marine vertebrates drives these ecosystems in a bottom-up manner (Bokhorst et al. 2019). However, these studies span large spatial scales, and a thorough understanding of heterogeneous environments like terrestrial Antarctica and their responses to environmental change requires studies at multiple spatial and temporal scales (Danis et al. 2020). Here, the dramatic fine-scale variation in midge abundance was best predicted by models that incorporate both abiotic and biotic influences from the environment. Thus, while historical work suggests that Antarctic ecosystems are abiotically regulated, our work and several other recent studies suggest that biotic interactions may be just as important, especially when considering fine-scale distributions. Surprisingly, in the best-supported model, substrate moisture was negatively associated with midge abundance, indicating that patterns of fine-scale distribution can be distinct from those of broad-scale distribution. Overall, these results indicate the importance of incorporating both abiotic and biotic components into species distribution models and the need to investigate species distributions on multiple spatial scales.

## Electronic supplementary material

Below is the link to the electronic supplementary material.

Supplementary file1 (DOCX 20 kb)

## References

[CR1] Barrett JE, Virginia RA, Lyons WB, McKnight DM, Priscu JC, Doran PT, Moorhead DL (2007). Biogeochemical stoichiometry of Antarctic dry valley ecosystems. J Geophys Res Biogeosci.

[CR2] Baust JG, Edwards JS (1979). Mechanisms of freezing tolerance in an Antarctic midge, *Belgica antarctica*.. Physiol Entomol.

[CR3] Bissett A, Abell GCJ, Brown M, Thrall PH, Bodrossy L, Smith MC, Richardsson AE (2014). Land-use and management practices affect soil ammonia oxidiser community structure, activity and connectedness. Soil Biol Biochem.

[CR4] Bokhorst S, Convey P, Aerts R (2019). Nitrogen inputs by marine vertebrates drive abundance and richness in Antarctic terrestrial ecosystems. Curr Biol.

[CR5] Bokhorst S, Convey P (2016). Impact of marine vertebrates on Antarctic terrestrial micro-arthropods. Antarct Sci.

[CR6] Caruso T, Hogg ID, Nielsen UN, Bottos EM, Lee CK, Hopkins DW, Wall DH (2019). Nematodes in a polar desert reveal the relative role of biotic interactions in the coexistence of soil animals. Commun Biol.

[CR8] Chong CW, Pearce DA, Convey P (2015). Emerging spatial patterns in Antarctic prokaryotes. Front Microbiol.

[CR9] Chown SL, Convey P (2016). Antarctic Entomology. Annu Rev Entomol.

[CR11] Convey P (1996). The influence of environmental characteristics on life history attributes of Antarctic terrestrial biota. Biol Rev.

[CR12] Convey P (1997). How are the life history strategies of Antarctic terrestrial invertebrates influenced by extreme environmental conditions?. J Therm Biol.

[CR13] Convey P (2011). Antarctic terrestrial biodiversity in a changing world. Polar Biol.

[CR14] Convey P, Block W (1996). Antarctic diptera: ecology, physiology and distribution. Eur J Entomol.

[CR16] Convey P, Gibson JAE, Hillenbrand CD, Hodgson DA, Pugh PJA, Smellie JL, Stevens MI (2008). Antarctic terrestrial life—challenging the history of the frozen continent?. Biol Rev.

[CR17] Convey P, Stevens MI (2007). Antarctic biodiversity. Science.

[CR19] Czechowski P, White D, Clarke L, McKay A, Cooper A, Stevens MI (2016). Age-related environmental gradients influence invertebrate distribution in the Prince Charles Mountains, East Antarctica.. R Soc Open Sci.

[CR20] Danis B, Van de Putte A, Convey P, Griffiths H, Linse K, Murray AE (2020). Antarctic biology: scale matters. Front Ecol Evol.

[CR25] Fabiszewski J, Wojtun B (2000). Chemical composition of some dominating plants in the maritime Antarctic tundra (King George Island). Bibl Lichenol.

[CR27] Guisan A, Thuiller W (2005). Predicting species distribution: offering more than simple habitat models. Ecol Lett.

[CR29] Hogg ID, Craig Cary S, Convey P, Newsham KK, O’Donnell AG, Adams BJ, Wall DH (2006). Biotic interactions in Antarctic terrestrial ecosystems: are they a factor?. Soil Biol Biochem.

[CR30] Hughes JB, Daily GC, Ehrlich PR (1997). Population diversity: its extent and extinction. Science.

[CR31] Ino Y, Nakatsubo T (1986). Distribution of carbon, nitrogen and phosphorus in a moss community-soil system developed on a cold desert in Antarctica. Ecol Res.

[CR32] Jacob J (1900). Diagnoses d’insects recueillis par l’expedition antartique Belge (parte chironomidae). Annu Soc Entomol Belg.

[CR34] Kennedy AD (1993). Water as a limiting factor in the Antarctic terrestrial environment: a biogeographical synthesis. Arct Alp Res.

[CR36] Lee CK, Laughlin DC, Bottos EM, Caruso T, Joy K, Barrett JE, Hopkins DW (2019). Biotic interactions are an unexpected yet critical control on the complexity of an abiotically driven polar ecosystem. Commun Biol.

[CR37] Lee RE, Denlinger DL (2014). Stress tolerance in a polyextremophile: the southernmost insect. Can J Zool.

[CR39] Lopez-Martinez G, Benoit JB, Rinehart JP, Elnitsky MA, Lee RE, Denlinger DL (2009). Dehydration, rehydration, and overhydration alter patterns of gene expression in the Antarctic midge, *Belgica antarctica*. J Comp Physiol B.

[CR41] Peckham V (1971). Notes on the chironomid midge *Belgica antarctica* Jacobs at Anvers Island in the maritime Antarctic. Pac Insects Monogr.

[CR42] Prietzel J, Prater I, Hurtarte LCC, Hrbáček F, Klysubun W, Mueller CW (2019). Site conditions and vegetation determine phosphorus and sulfur speciation in soils of Antarctica. Geochim Cosmochim Acta.

[CR43] Rico E, Quesada A (2013). Distribution and ecology of chironomids (diptera, chironomidae) on byers Peninsula maritime Antarctica. Antarct Sci.

[CR44] Ryan PG, Watkins BP (1989). The influence of physical factors and ornithogenic products on plant and arthropod abundance at an Inland Nunatak group in Antarctica. Polar Biol.

[CR45] Schlensog M, Green TGA, Schroeter B (2013). Life form and water source interact to determine active time and environment in cryptogams: an example from the maritime Antarctic. Oecologia.

[CR46] Sinclair BJ, Scott MB, Klok CJ, Terblanche JS, Marshall DJ, Reyers B, Chown SL (2006). Determinants of terrestrial arthropod community composition at Cape Hallett Antarctica. Antarct Sci.

[CR47] Sinclair BJ, Sjursen H (2001). Terrestrial invertebrate abundance across a habitat transect in Keble Valley, Ross Island Antarctica. Pedobiologia.

[CR49] Strong J (1967). Ecology of terrestrial arthropods at Palmer Station, Antarctic Peninsula. Antarct Res Ser.

[CR50] Stroup WW (2012). Generalized linear mixed models: modern concepts, methods and applications. CRC Press.

[CR51] Teets NM, Denlinger DL (2014). Surviving in a frozen desert: environmental stress physiology of terrestrial Antarctic arthropods. J Exp Biol.

[CR52] Usher MB, Booth RG (1986). Arthropod communities in a maritime Antarctic moss-turf habitat: multiple scales of pattern in the mites and Collembola. J Anim Ecol.

[CR53] Wasley J, Robinson SA, Lovelock CE, Popp M (2006). Climate change manipulations show Antarctic flora is more strongly affected by elevated nutrients than water. Glob Change Biol.

[CR54] Westgate MJ, Barton PS, Lane PW, Lindenmayer DB (2014). Global meta-analysis reveals low consistency of biodiversity congruence relationships. Nat Commun.

