# Supplementary material for: Environmental factors influencing fine-scale distribution of Antarctica’s only endemic insect
Source: Oecologia. 2020 Jul 29;194(4):529–39. doi: 10.1007/s00442-020-04714-9 (PMC7683470; doi:10.1007/s00442-020-04714-9)
Supplement: Supplementary file 1 — Supplementary file1 (DOCX 20 kb) [file 442_2020_4714_MOESM1_ESM.docx]

Table S1. Summary of all variables across islands. Mean ± standard error are reported. Midge, Collembola and Mite are reported in density/m^2^, Carbon and Nitrogen are percent total of the substrate measured, Moisture is percent content of water of substrate, Moss/Algae/Grass/Rocks are the proportion of the area of the plot, Bacteria is average abundance of OTUs, all elemental substances are reported in ng/mg of the substrate measured.

|  | Midge | Collembola | Mite | Carbon | Nitrogen | Moisture | Moss | Algae | Grass | Rocks | Bacteria | Al | Ca | Fe | K | Mg | Na | P | S |
| --- | --- | --- | --- | --- | --- | --- | --- | --- | --- | --- | --- | --- | --- | --- | --- | --- | --- | --- | --- |
| Amsler | 4230.8±  4303.8 | 21253.9±  43972.4 | 7.7±  27.7 | 44.1±  1.95 | 2.6±  0.7 | 0.4±  0.1 | 0.9±  0.1 | 0.02±  0.04 | 0.0±  0.0 | 0.1±  0.1 | 393.5±  66.2 | 1.9±  1.1 | 1.6±  0.5 | 0.9±  0.5 | 1.0±  0.7 | 0.9±  0.2 | 0.3±  0.07 | 1.8±  0.5 | 1.5±  0.4 |
| Christine | 7692.1± 472.4 | 25621.1±  73390.2 | 11828.9±  21202.6 | 25.3±  10.3 | 3.2±  1.0 | 0.3±  0.2 | 0.7±  0.2 | 0.03±  0.09 | 0.05±  0.08 | 0.3±  0.2 | 521.0±  148.7 | 3.1±  3.9 | 16.5±  15.9 | 1.8±  1.4 | 0.7±  0.7 | 1.0±  0.7 | 0.8±  0.7 | 10.2±  9.9 | 3.9±  3.3 |
| Cormorant | 7323.7±  1001.0 | 357.9±  865.3 | 3918.4±  7237.6 | 24.2±  13.3 | 2.9±  1.7 | 0.3±  0.1 | 0.4±  0.3 | 0.2±  0.2 | 0.08±  0.13 | 0.4±  0.2 | 682.2±  189.3 | 2.9±  2.9 | 21.3±  26.1 | 4.1±  5.8 | 0.9±  1.1 | 0.9±  1.1 | 0.8±  0.9 | 12.9±  14.8 | 4.5±  4.8 |
| Joubins 4 | 9937.5±  8637.9 | 8652.5±  14393.3 | 135.0±  340.3 | 43.2±  5.0 | 2.6±  0.7 | 0.3±  0.1 | 0.9±  0.1 | 0.0±  0.0 | 0.0±  0.0 | 0.1±  0.1 | 706.1±  178.5 | 2.3±  3.7 | 7.1±  13.6 | 1.6±  2.5 | 1.5±  0.7 | 1.5±  0.7 | 0.5±  0.4 | 5.1±  10.1 | 2.9±  2.3 |
| Torgerson | 6083.3±  5520.2 | 758.3±  2666.3 | 913.9±  1036.8 | 32.3±  7.7 | 4.1±  0.7 | 0.2±  0.1 | 0.2±  0.3 | 0.4±  0.3 | 0.04±  0.08 | 0.4±  0.2 | 643.2±  151.9 | 6.5±  5.7 | 36.4±  24.7 | 2.4±  1.4 | 3.7±  3.5 | 1.5±  0.6 | 1.5±  0.8 | 26.7±  18.3 | 5.9±  3.5 |
